# Autophagy and Its Effects: Making Sense of Double-Edged Swords

**DOI:** 10.1371/journal.pbio.1001967

**Published:** 2014-10-14

**Authors:** Andrew Thorburn

**Affiliations:** Department of Pharmacology, University of Colorado School of Medicine, Aurora, Colorado, United States of America

## Abstract

Autophagy delivers cellular material to lysosomes for recycling. This article discusses why both good and bad effects arise because of autophagy and argues that exploiting this knowledge can treat disease.

**Summary**

Autophagy is the mechanism by which cellular material is delivered to lysosomes and degraded. This process has become a major focus of biological and biomedical research with thousands of papers published each year and rapidly growing appreciation that autophagy affects many normal and pathological processes. However, as we learn more about this evolutionarily ancient process, we are discovering that autophagy's effects may work for both the good and the bad of an organism. Here, I discuss some of these context-dependent findings and how, as we make sense of them, we can try to apply our knowledge for practical purposes.

As I started on this essay, I googled “double-edged sword autophagy” and got pages of references to articles and books making the point that sometimes autophagy is good and sometimes it's bad. Most of the articles were linking the double-edged sword metaphor to diseases such as cancer, diabetes, infection, neurological disease, ischemic injury, etc. This implies that if we understand when autophagy is a good thing and when it's a bad thing, it will be feasible to improve treatment or prevention of these diseases by manipulating autophagy up or down. Attempts to do this in people are already underway. For example, we recently reported a deliberate attempt to inhibit autophagy in a child with a therapy-resistant brain tumor [1], and the first of dozens of ongoing Phase I and II clinical trials of autophagy inhibitors in cancer patients are starting to be reported [2]–[7]. Additionally, we know that we are often inadvertently manipulating autophagy when we treat people for various diseases. (This is because many currently used drugs affect autophagy—for example, cancer drugs often affect autophagy in addition to their intended targets [8]). If we understood how autophagy impacts on health and disease, could we improve disease treatment and prevention?

This essay will provide a personal (and very selective) view of these issues, but first I should define what I mean by “good,” “bad,” and “autophagy.” When I say “good” and “bad,” I mean with respect to a host organism. So, when considering the roles of autophagy in cancer or infection, “good” means good for the host, not for the tumor cell or infectious organism. And, the term “autophagy” will refer to macroautophagy, which is by far the best understood of the various types of autophagy (the others are chaperone mediated autophagy and microautophagy). Macroautophagy (for review, see [9]) depends on the coordinated actions of autophagy-related proteins (ATGs) that recruit membranes to form a double membrane vesicle called an autophagosome. The autophagosome engulfs cytoplasmic material, including proteins and organelles. The intact autophagosome then fuses with the lysosome, thus allowing lysosomal hydrolases to degrade the engulfed material and macromolecular precursors to be recycled for use in metabolism and to build new macromolecules.

## Autophagy

There are many excellent review articles on autophagy and its regulation, so I won't go into great detail about the process here. Two important points are needed to follow my argument. First, autophagy is a dynamic process whereby autophagosomes are formed, engulf their cargo (this can be selective, such that specific cargos are delivered to the autophagosome, or non-selective, whereby anything in the vicinity finds itself inside the autophagosome), and fuse with lysosomes, then the contents of the autophagosome are degraded. Completion of the whole process is termed autophagic flux, and its dynamic nature sometimes causes problems in interpretation of experimental data. For example, an increase in autophagosome number can be achieved either by making more (i.e., increasing autophagy) or by degrading fewer of them (i.e., a decrease in autophagy). Some papers do not adequately distinguish between these two opposing possibilities, and it should be borne in mind that some conclusions about apparently competing effects of autophagy may be misinterpretations in which someone thinks they were getting more autophagy when in fact they were getting less, or vice versa. Second, distinct regulators that can be inhibited with pharmacological agents or genetic interventions (knockouts, knockdowns, and expression of dominant negative mutants) control each step in the process. Thus, if you want to inhibit autophagy at early steps, you might knock down BECN1, a scaffolding protein that is critical for the initial steps in autophagosome formation, whereas to prevent elongation of the autophagosomal membrane, you might target ATG7, which is required for protein conjugations needed to elongate the membrane. To block autophagy at later steps one might target the lysosome with a drug like chloroquine (CQ) or hydroxychloroquine (HCQ) that prevents lysosomal fusion with the autophagosome. An important caveat to bear in mind is that these interventions are not necessarily specific. For example, all the clinical trials mentioned above are testing the idea that autophagy inhibition by HCQ will increase tumor chemosensitivity to other anticancer drugs. However, tumor cells can also be chemosensitized by CQ through autophagy-independent mechanisms [10]. Similarly, although knockout or knockdown of ATGs can be a highly effective way to inhibit autophagy, many, perhaps all, ATGs have other functions as well [11]. This means that if your ATG knockout had a phenotype, it could be due to other functions of that protein rather than blocking autophagy.

With these definitions and caveats noted, I will try to make two points. (1) Autophagy is indeed both good and bad in different circumstances. (2) By understanding the mechanisms that underlie these divergent effects, we can start to see how we can apply this knowledge in a rational way to get outcomes that we want, such as effective treatments for the diseases where autophagy plays a role.

## Autophagy and Bacterial Infection

In the last decade, there has been much work on the role of autophagy in pathogen infection and antimicrobial immunity (see [12],[13] for recent reviews). Autophagy's roles in this area are complicated, involving direct degradation of microorganisms (a process called xenophagy, in which bacteria or viruses are engulfed in autophagosomes, then degraded by the lysosomal enzymes) as well as regulation of proinflammatory signaling, antigen presentation, adaptive immune responses, and secretion of immune mediators [12]. Even if we just consider what one might think of as a simple case, i.e. engulfment of bacteria by the autophagy machinery, sometimes this is good, and sometimes it's bad. The good side of xenophagy is obvious—pathogenic bacteria are selectively targeted to autophagosomes and degraded, providing a mechanism by which infection is reduced [13]. The importance of this for overall health of the host is nicely demonstrated by a recent study from Eileen White's lab, describing the first mouse with inducible full body knockout of autophagy. Adult mice in which autophagy is efficiently inhibited in all cells in the body (due to knockout of *Atg7*) display a marked two-step pattern of lethality due to autophagy inhibition. Some animals die straight away because they succumb to *Streptococcus* infection [14]. This makes sense because autophagic degradation of *Streptococcus* is known to occur [15]. Thus, autophagy and/or xenophagy is good for the mouse in this case. (The remaining mice die more slowly due to neurodegeneration. In this essay I'm going to steer away from autophagy in the brain, but the reader should be aware that this phenotype fits well with known protective effects of autophagy against neuronal cell death.) But, autophagy is not always a good thing when it comes to bacterial infection. Other intracellular bacteria have to be engulfed by the autophagy machinery in order to replicate. For example, *Brucella*, the bacteria responsible for brucellosis, use the core autophagy machinery to create an intracellular compartment [16] that is needed to complete the *Brucella* lifecycle and cell-to-cell spreading. Thus, the autophagy process has been used in this case to promote bacterial infection and is therefore bad for the host.

The important point is this: when thinking about how we should manipulate autophagy in order to deal with intracellular bacterial infection, it is clear that one can't just say autophagy is good or it's bad. For some bacteria, autophagy is how our cells kill them, for others it's how they replicate and spread throughout a host. Similar effects are seen with viruses, too—some rely on the autophagy machinery to replicate; others are blocked by autophagy. So, we can't just say that more or less autophagy would be what we should aim for to treat or prevent infectious disease; we need to understand how autophagy impacts the infectious process and design manipulations accordingly.

## Autophagy and Cancer

For some time, we've been talking about context-dependent effects and “doubled-edged swords” when it comes to autophagy and cancer [17]–[21]. Broadly speaking, autophagy may protect against cancer development, but be important for cancer progression and treatment. Thus, we might want to boost autophagy to prevent cancer but inhibit it as part of a cancer treatment strategy.

Evidence in favor of autophagy functioning as a tumor suppressor comes mostly from discoveries that inactivation of certain autophagy regulators causes cancer in mice and that some human tumors display loss of these regulators. For example, allelic loss of *Becn1* causes increased tumor development in mice [22],[23], and the *BECN1* gene is deleted in human tumors [24]. These data appear to suggest that autophagy suppresses development of malignant tumors. A slightly different interpretation comes from deletion of other essential autophagy genes. For example, systemic mosaic deletion of the *Atg5* or *Atg7* genes in mice doesn't lead to true malignancy. Instead, only benign adenomas are found in these mice, and only in the liver [25]. This result implies that autophagy may be capable of suppressing only the very earliest stages of tumor development, not the appearance of true malignant tumors. It should also be noted that a recent analysis of thousands of human tumors found no evidence of specific *BECN1* deletions or somatic mutations but instead concluded that the apparent loss of *BECN1* in human cancer is most likely due to deletion of the adjacent *BRCA1* gene, which is a well-known tumor suppressor [26]. I think there are two important points to consider here. First, we need to be careful not to jump to the conclusion that just because a gene is important for controlling autophagy and also for suppressing cancer, that this necessarily means that autophagy is important for suppressing cancer. A potential explanation for the *Becn1* gene deletion effects in mice compared with the milder tumorigenic effects of *Atg5* or *Atg7* deletions might be that other functions of the BECN1 protein independent of its role in autophagy are causing the tumor phenotype. Second, even if autophagy is preventing cancer development, the results of Takamura et.al. show that there may be marked differences in different tissues [25] —sometimes it's good, sometimes not.

After a malignant tumor is formed, autophagy's roles are still complicated. A great deal of work has focused on how autophagy affects response to cancer therapy [8] and as noted at the beginning of this essay, this is where we are furthest along in clinical application of autophagy manipulation. Here I want to focus on one particular complication: different tumor cells behave differently when autophagy is inhibited. In a recent paper [27], my group reported that different breast cancer cells behave quite differently when autophagy is inhibited either genetically or pharmacologically. For some cells, autophagy inhibition has little effect or merely slows their growth a bit. For other breast cancer cells, autophagy is essential for survival even in the absence of any added stress. For these cells, simply inhibiting autophagy is sufficient to cause the tumor cells to die. This suggests that some tumor cells are dependent on autophagy, but others are not. Moreover, we also found that it is only for the autophagy-dependent cells that combining autophagy inhibitor with another anticancer drug is synergistic. In fact, such combinations in autophagy-independent tumor cells were more likely to be antagonistic than to show synergy. Similar results were found when we tried to inhibit autophagy along with other anticancer drugs in brain cancer cells; only autophagy-dependent cells displayed synergy [1]. Thus, when it comes to treatment with other drugs, autophagy in some tumors is good and in others, bad because if we inhibit autophagy at the same time as treating with other drugs, this can increase tumor cell death only in some tumor cells, while in others it may actually lead to less tumor cell death. This work from our lab complements a number of prior papers from other groups showing that certain tumors, most notably those where the RAS pathway is mutated, are especially sensitive to autophagy inhibition [14],[28]–[31]. In these studies, too, it is clear that some tumor cells rely more on autophagy than others. It should be noted that in our breast cell study we mostly examined tumor lines with no RAS pathway mutations. This means that mutations in this pathway are not the only way that tumors may become highly dependent upon autophagy. However, the general concept remains: autophagy has competing effects in different contexts. This work highlights the importance of identifying the mechanisms by which tumor cells acquire autophagy-dependence or autophagy-independence and determining whether these characteristics are stable—e.g., if a tumor cell that is not initially autophagy-dependent can become dependent as it evolves in response to selective pressures such as drug treatment.

## Autophagy and Cell Death

To end this essay, let's consider what might seem like a much simpler case of a single fundamental biological response—cell death. Does autophagy promote or inhibit cell death? There has been much confusion and discussion over the years about whether autophagy can cause a form of programmed cell death [32],[33] and whether it prevents or promotes apoptosis [34]. The reason I want to finish up by considering how autophagy can be both good and bad when it comes to cell death is that I think our efforts to understand how such apparently divergent responses come about may serve as a model for how to make sense of competing effects in other situations too.

A recent study from our group [35] provided a surprising example of competing effects of autophagy. We developed a method to separate living cells from a genetically homogeneous population based on differences in their level of autophagic flux and discovered that even under conditions in which there is no external inducer of autophagy, some cells in a population are undergoing much more autophagy than others. This variation is transient, such that cells that are doing high autophagy and those that are doing low autophagy remain high or low for 4–8 hours, but over time the population distribution reverts to that of the original population. The interesting discovery was that high or low autophagy predicted the subsequent likelihood of the cell dying when treated with an apoptotic stimulus. So, when we purified the highest autophagy quartile and the lowest autophagy quartile from the total cell population, they differed markedly in their sensitivity to subsequent treatment with an apoptotic stimulus. Surprisingly, however, high autophagy cells were more sensitive than the starting population to one apoptosis inducer but less sensitive to another. This was particularly surprising because the two apoptosis stimuli that we used (Fas Ligand and TRAIL) work in very similar ways. They are both death receptor agonists that activate well-understood signaling pathways involving the same sets of downstream signaling proteins to induce apoptosis. How can we make sense of this? We isolate cells from a population based on the amount of autophagy they are doing. This amount of autophagy predicts whether or not they will die when treated hours later with an apoptotic stimulus, but two very similar apoptotic stimuli behave in completely opposite ways in the same isolated cells. To add further confusion, these effects were cell-type specific. In some cells, high autophagy made them more sensitive to Fas Ligand–induced apoptosis but less sensitive to TRAIL while, in other cells, high autophagy cells were less sensitive to both Fas Ligand and TRAIL. What we have here is a cell-level example of autophagy sometimes being good and sometimes bad. The explanation is that a negative regulator of Fas signaling called FAP1 is selectively degraded by autophagy. The important point is that FAP1 doesn't affect TRAIL. Moreover, FAP1 is expressed only in some cells, and it is only these cells in which autophagy promotes Fas Ligand-induced apoptosis. FAP1 degradation therefore explains why autophagy sometimes promotes Fas-induced apoptosis and sometimes doesn't, and why autophagy only promotes Fas and not TRAIL-induced apoptosis in this case. If FAP1 is not expressed in a cell, it can't be degraded by autophagy, and, even if it is expressed, FAP1 degradation will only affect signaling pathways that FAP1 regulates. This allows us to make sense of our otherwise confusing results—the opposing effects and cell-type specificity of autophagy's effects are explained by what autophagy is degrading. Another paper explained at least part of the reason why autophagy was protective against TRAIL—this was due to autophagy's ability to selectively regulate the levels of a different apoptosis regulator, PUMA, which is needed to increase the efficiency of apoptosis [36]. Therefore, in one case autophagic degradation of a negative regulator of a cell death pathway makes autophagy a promoter of apoptosis, while in other contexts autophagy is causing a reduction in a positive regulator of apoptosis and this makes the process protective against cell death. What does this mean for other apparently contradictory effects of autophagy—e.g., why autophagy protects tumor cells against chemotherapy in some circumstances but not others or is critically important for survival in only some tumor cells? I suspect that this too will be due to differences in what autophagy is degrading under these specific circumstances. This is a pretty trivial and straightforward hypothesis—the effects of a process that degrades cellular material depend on what it degrades. The difficult thing is testing the hypothesis and then using this knowledge to make predictions about autophagy being good or bad. To test the hypothesis we need to find out what gets degraded under different circumstances—e.g., tumor cells in which autophagy is critical, compared to those in which it is not, and tissues in which autophagy protects against cancer development and those in which it doesn't. A recent paper from the Kimmelman and Harper laboratories suggests a way to identify selectively degraded proteins [37]. In this paper the authors developed a method for quantitative proteomic analysis of autophagy cargos and, using this approach, discovered another example whereby selective autophagy of specific proteins (in their case, ferritin) is achieved. I think this is an important advance because it will allow us to identify more substrates of autophagy and discover under what circumstances they are or are not targeted for degradation. This may be the key to determining when autophagy is good or bad and then applying that knowledge. For example, if we can identify the important substrates whose degradation makes certain tumor cells more or less dependent on autophagy, this may provide a way to decide which tumors should or shouldn't be treated with autophagy inhibitors.

As is often the case, the promise of manipulation of a biological process relies on understanding how the process really works. Also, we need basic mechanistic understanding of biological processes if we want to apply them for clinical benefit or other practical application. Given the rapid progress in understanding autophagy and what it does, I think such applications are becoming more feasible.
